# Supplementary material for: Molecular Tracing of SARS-CoV-2 in Italy in the First Three Months of the Epidemic
Source: Viruses. 2020 Jul 24;12(8):798. doi: 10.3390/v12080798 (PMC7472216; doi:10.3390/v12080798)
Supplement: Supplementary file 1 [file viruses-12-00798-s001.zip › Table S1.pdf]

Table S1. Data of Italian Patients characterized in the present study.

Sample Code	Region	City	Province	Sample Date	Sample Type	Gender	Age
EC	Lombardy	Milano	MI	2020-02-24	Sputum	M	62
PD	Lombardy	Milano	MI	2020-02-24	Isolate	M	43
SV	Lombardy	Milano	MI	2020-02-24	NP swab	F	36
VL	Lombardy	Milano	MI	2020-02-24	Sputum	F	72
PL	Lombardy	Milano	MI	2020-02-24	Sputum	M	60
FLC	Lombardy	Milano	MI	2020-02-24	Sputum	F	61
RM	Lombardy	Milano	MI	2020-03-11	Sputum	M	76
BA	Lombardy	Milano	MI	2020-03-13	Sputum	M	59
PAD	Lombardy	Milano	MI	2020-03-17	Sputum	M	43
MM	Lombardy	Milano	MI	2020-03-16	Sputum	M	63
RS	Lombardy	Milano	MI	2020-03-17	Sputum	F	47
BE	Lombardy	Milano	MI	2020-03-18	Sputum	M	78
MC	Lombardy	Milano	MI	2020-03-11	Sputum	F	NA
C96	Lombardy	Milano	MI	2020-03-04	Sputum	M	31
BP1_MI	Lombardy	Milano	MI	2020-02-28	Isolate	M	58
5_MI	Lombardy	Milano	MI	2020-04-27	Isolate	F	80
6_MI	Lombardy	Cambiago	MI	2020-04-27	Isolate	M	91
10_MI	Lombardy	Bresso	MI	2020-04-28	Isolate	M	95
FS75	Lombardy	Chiari	BS	2020-03-09	Isolate	F	45
GC39	Lombardy	Brescia	BS	2020-03-09	Isolate	M	81
GC65	Lombardy	Manerbio	BS	2020-03-09	Isolate	F	55
MS58	Lombardy	Desenzano	BS	2020-03-09	Isolate	M	62
TS	Veneto	Padova	PD	2020-03-24	NP swab	M	84
PD004	Veneto	Grantorto	PD	2020-03-04	NP swab	M	73
PD006	Veneto	Vigodarzere	PD	2020-03-05	NP swab	M	92
PD010	Veneto	Bergamo	PD	2020-03-08	NP swab	M	49
PD009	Veneto	Padova	PD	2020-03-23	NP swab	F	43
PD002	Veneto	Padova	PD	2020-03-09	NP swab	M	93
PD013	Veneto	Padova	PD	2020-03-11	NP swab	F	22
PD017	Veneto	Ponte San Nicolò	PD	2020-03-16	NP swab	M	78
DG	Veneto	Limena	PD	2020-03-31	NP swab	M	69
PD008	Veneto	Campodarsego	PD	2020-03-13	NP swab	M	76
UnivPM02	Marche	Fano	PS	2020-02-28	Isolate	F	47
UnivPM03	Marche	Fano	PS	2020-02-28	Isolate	F	52
UnivP04	Marche	Fano	PS	2020-02-26	Isolate	M	80
UnivPM05	Marche	Fano-Centinarola	PS	2020-03-04	Isolate	M	52
UnivPM06	Marche	Fano	PS	2020-03-04	Isolate	M	44
UnivPM7	Marche	Pesaro-Montelabbate	PS	2020-03-08	Isolate	F	31
UnivPM8	Marche	Pesaro	PS	2020-03-09	Isolate	F	57
UnivPM9	Marche	Ancona	AN	2020-03-09	Isolate	F	65
UnivPM10	Marche	Macerata-Corridonia	MC	2020-03-18	Isolate	F	63
UNISI1	Tuscany	Sovicille	SI	2020-03-26	NP swab	M	61
UNISI4	Tuscany	Siena	SI	2020-03-26	NP swab	F	50
UNISI6	Tuscany	Siena	SI	2020-03-29	NP swab	M	69
UNISI7	Tuscany	Arezzo	AR	2020-03-12	NP swab	M	63
UNISI13	Tuscany	Arezzo	AR	2020-03-12	NP swab	M	62
UNISI14	Tuscany	Arezzo	AR	2020-03-12	NP swab	F	41
UNISI24	Tuscany	Grosseto	GR	2020-03-18	NP swab	M	66
UNISI37	Tuscany	Monteriggioni	SI	2020-03-27	NP swab	M	56
UNISI38	Tuscany	Sarteano	SI	2020-03-27	NP swab	M	42
UNISI43	Tuscany	Monteriggioni	SI	2020-03-26	NP swab	F	51
UNISI46	Tuscany	Monteriggioni	SI	2020-03-25	NP swab	F	51
UNISI51	Tuscany	Arezzo	AR	2020-03-01	NP swab	F	42
UNISI52	Tuscany	Grosseto	GR	2020-02-27	NP swab	M	22
C4	Lombardy	Cremona	CR	2020-03-13	NP swab	M	72
C12	Lombardy	Cremona	CR	2020-03-14	NP swab	M	60
C13	Lombardy	Cremona	CR	2020-03-26	NP swab	M	24
B6	Lombardy	Bergamo	BG	2020-03-29	BAL	NA	NA
B9	Lombardy	Bergamo	BG	2020-03-29	BAL	NA	NA
