# Supplementary material for: Molecular Tracing of SARS-CoV-2 in Italy in the First Three Months of the Epidemic
Source: Viruses. 2020 Jul 24;12(8):798. doi: 10.3390/v12080798 (PMC7472216; doi:10.3390/v12080798)
Supplement: Supplementary file 1 [file viruses-12-00798-s001.zip › Table S2.pdf]

Table S2. Accession IDs, sampling dates and location of sequences included in the dataset.

Accession ID	Sampling date	Location
EPI_ISL_413570	2020-02-28	NL
EPI_ISL_426883	2020-03-27	CZ
EPI_ISL_426884	2020-03-27	CZ
EPI_ISL_426888	2020-03-31	CZ
EPI_ISL_426889	2020-04-02	CZ
EPI_ISL_426890	2020-03-26	CZ
EPI_ISL_426892	2020-03-27	CZ
EPI_ISL_426896	2020-03-28	CZ
EPI_ISL_426897	2020-03-29	CZ
EPI_ISL_436201	2020-03-18	ES
EPI_ISL_436208	2020-03-22	ES
EPI_ISL_436217	2020-03-13	ES
EPI_ISL_424342	2020-03-07	IT
EPI_ISL_424343	2020-03-23	IT
EPI_ISL_424344	2020-03-04	IT
EPI_ISL_424366	2020-03-17	TR
EPI_ISL_424378	2020-03-19	IS
EPI_ISL_436339	2020-03-28	ES
EPI_ISL_436386	2020-03-17	ES
EPI_ISL_413603	2020-03-03	FI
EPI_ISL_413604	2020-03-03	FI
EPI_ISL_413647	2020-03-01	PT
EPI_ISL_436368	2020-03-25	ES
EPI_ISL_436372	2020-03-16	ES
EPI_ISL_436379	2020-03-18	ES
EPI_ISL_436396	2020-03-22	ES
EPI_ISL_437308	2020-03-25	TR
EPI_ISL_437309	2020-03-26	TR
EPI_ISL_437312	2020-03-25	TR
EPI_ISL_437325	2020-03-19	TR
EPI_ISL_437327	2020-03-19	TR
EPI_ISL_403928	2020-01-01	CN
EPI_ISL_403929	2019-12-30	CN
EPI_ISL_403930	2019-12-30	CN
EPI_ISL_403931	2019-12-30	CN
EPI_ISL_414631	2020-03-04	FR
EPI_ISL_437301	2020-04-17	AT
EPI_ISL_437302	2020-04-17	AT
EPI_ISL_437303	2020-04-17	AT
EPI_ISL_437332	2020-03-18	TR
EPI_ISL_413489	2020-03-03	IT
EPI_ISL_414625	2020-02-26	FR
EPI_ISL_414638	2020-03-04	FR
EPI_ISL_414642	2020-03-08	FI
EPI_ISL_414643	2020-03-07	FI
EPI_ISL_425464	2020-03-11	GB
EPI_ISL_437435	2020-04-04	RS
EPI_ISL_424703	2020-03-15	SE
EPI_ISL_413997	2020-02-26	CH
EPI_ISL_413999	2020-02-27	CH
EPI_ISL_424638	2020-04-03	BE
EPI_ISL_434010	2020-04-14	GB
EPI_ISL_435409	2020-03-25	HU
EPI_ISL_422156	2020-03-28	GB
EPI_ISL_435406	2020-03-22	HU
EPI_ISL_435411	2020-03-27	HU
EPI_ISL_435412	2020-03-30	HU
EPI_ISL_435413	2020-03-30	HU
EPI_ISL_435414	2020-03-30	HU
EPI_ISL_435415	2020-03-30	HU
EPI_ISL_435416	2020-03-27	HU
EPI_ISL_435417	2020-03-30	HU
EPI_ISL_435419	2020-03-20	HU
EPI_ISL_435420	2020-03-23	HU
EPI_ISL_435421	2020-03-25	HU
EPI_ISL_435429	2020-03-28	HU
EPI_ISL_435431	2020-03-25	HU
EPI_ISL_436716	2020-04-14	RU
EPI_ISL_436718	2020-03-19	IT
EPI_ISL_436719	2020-03-20	IT
EPI_ISL_436720	2020-03-20	IT
EPI_ISL_436721	2020-03-20	IT
EPI_ISL_436722	2020-03-20	IT
EPI_ISL_436723	2020-03-20	IT
EPI_ISL_436724	2020-03-21	IT
EPI_ISL_436725	2020-04-27	IT
EPI_ISL_436726	2020-04-27	IT
EPI_ISL_436727	2020-04-27	IT
EPI_ISL_436728	2020-04-27	IT
EPI_ISL_436729	2020-04-27	IT
EPI_ISL_436730	2020-04-27	IT
EPI_ISL_436731	2020-04-26	IT
EPI_ISL_436732	2020-04-27	IT
EPI_ISL_424491	2020-03-20	IS
EPI_ISL_435145	2020-03-24	IT
EPI_ISL_435146	2020-04-07	IT
EPI_ISL_435147	2020-04-08	IT
EPI_ISL_435148	2020-04-08	IT
EPI_ISL_435149	2020-04-08	IT
EPI_ISL_435150	2020-04-08	IT
EPI_ISL_435151	2020-04-08	IT
EPI_ISL_435152	2020-04-09	IT
EPI_ISL_435153	2020-04-09	IT
EPI_ISL_435154	2020-04-09	IT
EPI_ISL_435155	2020-04-09	IT
EPI_ISL_424415	2020-03-19	IS
EPI_ISL_424434	2020-03-19	IS
EPI_ISL_424586	2020-03-27	IS
EPI_ISL_424606	2020-03-28	IS
EPI_ISL_424556	2020-03-29	IS
EPI_ISL_414020	2020-02-27	CH
EPI_ISL_426051	2020-03-20	CZ
EPI_ISL_427356	2020-04-06	BE
EPI_ISL_427367	2020-04-07	BE
EPI_ISL_428681	2020-03-11	ES
EPI_ISL_428684	2020-03-27	ES
EPI_ISL_417963	2020-03-12	ES
EPI_ISL_417997	2020-03-07	PT
EPI_ISL_427318	2020-04-03	RU
EPI_ISL_402129	2019-12-30	CN
EPI_ISL_402130	2019-12-30	CN
EPI_ISL_402132	2019-12-30	CN
EPI_ISL_415481	2020-03-08	NL
EPI_ISL_415485	2020-03-12	NL
EPI_ISL_415489	2020-03-13	NL
EPI_ISL_416751	2020-03-05	FR
EPI_ISL_416754	2020-03-06	FR
EPI_ISL_417921	2020-03-01	IT
EPI_ISL_417922	2020-02-28	IT
EPI_ISL_417923	2020-03-04	IT
EPI_ISL_428712	2020-03-17	TR
EPI_ISL_428714	2020-03-18	TR
EPI_ISL_428715	2020-03-18	TR
EPI_ISL_428718	2020-03-19	TR
EPI_ISL_428719	2020-03-21	TR
EPI_ISL_428720	2020-03-21	TR
EPI_ISL_428721	2020-03-21	TR
EPI_ISL_429968	2020-02-21	FR
EPI_ISL_402119	2019-12-30	CN
EPI_ISL_402120	2020-01-01	CN
EPI_ISL_402121	2019-12-30	CN
EPI_ISL_402123	2019-12-24	CN
EPI_ISL_415454	2020-02-28	CN
EPI_ISL_415457	2020-02-29	CN
EPI_ISL_417785	2020-03-12	IS
EPI_ISL_429771	2020-03-23	LU
EPI_ISL_429774	2020-03-23	LU

EPI_ISL_429777	2020-03-24	LU
EPI_ISL_415156	2020-03-01	BE
EPI_ISL_415159	2020-02-29	BE
EPI_ISL_416483	2020-02-26	ES
EPI_ISL_416487	2020-03-04	ES
EPI_ISL_416488	2020-03-03	PL
EPI_ISL_417704	2020-03-10	IS
EPI_ISL_417747	2020-03-11	IS
EPI_ISL_417791	2020-03-12	IS
EPI_ISL_429767	2020-03-25	LU
EPI_ISL_429799	2020-03-30	LU
EPI_ISL_416426	2020-03-17	HU
EPI_ISL_417733	2020-03-16	IS
EPI_ISL_417765	2020-02-27	IS
EPI_ISL_429731	2020-03-31	LU
EPI_ISL_429736	2020-03-29	LU
EPI_ISL_429871	2020-03-23	TR
EPI_ISL_430852	2020-03-11	SE
EPI_ISL_416507	2020-03-05	FR
EPI_ISL_417829	2020-03-16	IS
EPI_ISL_417834	2020-03-16	IS
EPI_ISL_417836	2020-03-16	IS
EPI_ISL_417877	2020-03-06	SK
EPI_ISL_417878	2020-03-07	SK
EPI_ISL_417879	2020-03-06	SK
EPI_ISL_417880	2020-03-08	SK
EPI_ISL_429863	2020-03-22	TR
EPI_ISL_429864	2020-03-22	TR
EPI_ISL_429867	2020-03-17	TR
EPI_ISL_430847	2020-02-27	SE
EPI_ISL_430848	2020-03-11	SE
EPI_ISL_430849	2020-03-11	SE
EPI_ISL_414441	2020-03-03	NL
EPI_ISL_414471	2020-03-05	NL
EPI_ISL_414477	2020-03-01	CZ
EPI_ISL_437199	2020-03-31	AT
EPI_ISL_414468	2020-03-06	NL
EPI_ISL_415703	2020-03-01	CH
EPI_ISL_415704	2020-03-04	CH
EPI_ISL_415707	2020-03-08	CH
EPI_ISL_425130	2020-03-21	DE
EPI_ISL_425139	2020-03-19	DE
EPI_ISL_425140	2020-03-20	DE
EPI_ISL_437273	2020-03-30	DE
EPI_ISL_437286	2020-04-02	DE
EPI_ISL_414505	2020-02-27	DE
EPI_ISL_414584	2020-03-02	IE
EPI_ISL_414585	2020-03-03	IE
EPI_ISL_414586	2020-03-03	IE

EPI_ISL_414587	2020-03-03	IE
EPI_ISL_425223	2020-03-17	ES
EPI_ISL_425228	2020-03-22	CZ
EPI_ISL_437203	2020-04-06	AT
EPI_ISL_437210	2020-03-07	DE
EPI_ISL_437211	2020-03-12	DE
EPI_ISL_437218	2020-03-12	DE
EPI_ISL_437221	2020-04-06	DE
EPI_ISL_437227	2020-04-13	DE
EPI_ISL_437231	2020-03-25	DE
EPI_ISL_437236	2020-03-22	DE
EPI_ISL_437237	2020-03-23	DE
EPI_ISL_437238	2020-03-24	DE
EPI_ISL_437243	2020-04-11	DE
EPI_ISL_415525	2020-03-12	NL
EPI_ISL_426285	2020-03-30	LV
EPI_ISL_426286	2020-03-30	LV
EPI_ISL_426287	2020-03-30	LV
EPI_ISL_426288	2020-03-30	LV
EPI_ISL_426289	2020-03-30	LV
EPI_ISL_428853	2020-03-30	IT
EPI_ISL_428854	2020-03-30	IT
EPI_ISL_428883	2020-03-20	RU
EPI_ISL_428888	2020-03-20	RU
EPI_ISL_428893	2020-03-23	RU
EPI_ISL_428895	2020-03-24	RU
EPI_ISL_428897	2020-03-24	RU
EPI_ISL_415637	2020-03-04	GB
EPI_ISL_425052	2020-03-25	BE
EPI_ISL_425059	2020-03-24	BE
EPI_ISL_426379	2020-03-29	SI
EPI_ISL_428865	2020-03-11	RU
EPI_ISL_428958	2020-04-07	LU
EPI_ISL_437016	2020-03-26	DK
EPI_ISL_437031	2020-03-27	DK
EPI_ISL_437089	2020-03-23	LV
EPI_ISL_437092	2020-03-20	LV
EPI_ISL_437093	2020-03-22	LV
EPI_ISL_437094	2020-03-23	LV
EPI_ISL_437095	2020-03-23	LV
EPI_ISL_437096	2020-03-23	LV
EPI_ISL_413019	2020-02-26	CH
EPI_ISL_413022	2020-02-29	CH
EPI_ISL_426358	2020-03-19	CZ
EPI_ISL_426359	2020-03-17	CZ
EPI_ISL_428925	2020-03-28	PL
EPI_ISL_428926	2020-03-28	PL
EPI_ISL_428927	2020-03-28	PL
EPI_ISL_428928	2020-03-30	PL

EPI_ISL_428930	2020-03-29	PL
EPI_ISL_428931	2020-03-28	PL
EPI_ISL_428932	2020-03-28	PL
EPI_ISL_429116	2020-03-15	SE
EPI_ISL_429120	2020-03-11	SE
EPI_ISL_429136	2020-03-04	SE
EPI_ISL_430100	2020-04-14	RU
EPI_ISL_430110	2020-04-15	RU
EPI_ISL_430111	2020-04-15	RU
EPI_ISL_418402	2020-03-14	FI
EPI_ISL_418403	2020-03-14	FI
EPI_ISL_418404	2020-03-14	FI
EPI_ISL_418405	2020-03-14	FI
EPI_ISL_418406	2020-03-14	FI
EPI_ISL_418407	2020-03-14	FI
EPI_ISL_418409	2020-03-14	FI
EPI_ISL_418432	2020-03-18	FR
EPI_ISL_418580	2020-03-08	IE
EPI_ISL_418581	2020-03-08	IE
EPI_ISL_418582	2020-03-10	IE
EPI_ISL_418583	2020-03-10	IE
EPI_ISL_420899	2020-03-11	DE
EPI_ISL_429230	2020-03-24	IT
EPI_ISL_429231	2020-03-24	IT
EPI_ISL_429232	2020-03-24	IT
EPI_ISL_429233	2020-03-24	IT
EPI_ISL_429234	2020-03-24	IT
EPI_ISL_429235	2020-03-24	IT
EPI_ISL_429236	2020-03-28	IT
EPI_ISL_430076	2020-04-10	RU
EPI_ISL_430084	2020-04-09	RU
EPI_ISL_430087	2020-04-10	RU
EPI_ISL_430090	2020-04-10	RU
EPI_ISL_430092	2020-04-14	RU
EPI_ISL_430095	2020-04-14	RU
EPI_ISL_418516	2020-03-06	IE
EPI_ISL_429213	2020-03-16	CH
EPI_ISL_429286	2020-03-11	DK
EPI_ISL_429288	2020-03-11	DK
EPI_ISL_429295	2020-03-13	DK
EPI_ISL_429297	2020-03-14	DK
EPI_ISL_432805	2020-04-02	GB
EPI_ISL_406597	2020-01-23	FR
EPI_ISL_418548	2020-03-06	IE
EPI_ISL_419562	2020-02-29	LU
EPI_ISL_429199	2020-03-22	CH
EPI_ISL_429200	2020-03-19	CH
EPI_ISL_429207	2020-04-03	CH
EPI_ISL_429220	2020-04-06	CH

EPI_ISL_429226	2020-03-17	IT
EPI_ISL_429227	2020-03-17	IT
EPI_ISL_429228	2020-03-20	IT
EPI_ISL_429229	2020-03-21	IT
EPI_ISL_418228	2020-03-12	FR
EPI_ISL_418247	2020-02-26	ES
EPI_ISL_418255	2020-03-14	IT
EPI_ISL_418256	2020-03-14	IT
EPI_ISL_418257	2020-03-17	IT
EPI_ISL_418258	2020-03-14	IT
EPI_ISL_418259	2020-03-14	IT
EPI_ISL_418260	2020-03-16	IT
EPI_ISL_418261	2020-03-17	IT
EPI_ISL_418263	2020-03-18	GR
EPI_ISL_418264	2020-03-18	GR
EPI_ISL_418265	2020-03-18	GR
EPI_ISL_418277	2020-03-03	CH
EPI_ISL_419571	2020-03-06	LU
EPI_ISL_419572	2020-03-10	LU
EPI_ISL_419576	2020-03-17	LU
EPI_ISL_419579	2020-03-14	LU
EPI_ISL_419580	2020-03-12	LU
EPI_ISL_420541	2020-03-05	SI
EPI_ISL_420563	2020-03-18	IT
EPI_ISL_420564	2020-03-19	IT
EPI_ISL_420565	2020-03-19	IT
EPI_ISL_420566	2020-03-19	IT
EPI_ISL_420567	2020-03-21	IT
EPI_ISL_420568	2020-03-23	IT
EPI_ISL_420569	2020-03-23	IT
EPI_ISL_420583	2020-03-23	IT
EPI_ISL_420592	2020-03-23	IT
EPI_ISL_419546	2020-03-15	DE
EPI_ISL_417018	2020-03-14	BE
EPI_ISL_418183	2020-03-17	HU
EPI_ISL_419671	2020-03-19	AT
EPI_ISL_419673	2020-03-22	AT
EPI_ISL_419674	2020-03-24	AT
EPI_ISL_419686	2020-03-09	ES
EPI_ISL_418386	2020-03-13	FI
EPI_ISL_418387	2020-03-13	FI
EPI_ISL_418388	2020-03-13	FI
EPI_ISL_418389	2020-03-13	FI
EPI_ISL_418390	2020-03-13	FI
EPI_ISL_418392	2020-03-13	FI
EPI_ISL_418398	2020-03-13	FI
EPI_ISL_419654	2020-03-03	AT
EPI_ISL_419655	2020-02-26	AT
EPI_ISL_419656	2020-02-26	AT

EPI_ISL_419659	2020-03-10	AT
EPI_ISL_419660	2020-03-13	AT
EPI_ISL_419661	2020-03-10	AT
EPI_ISL_419662	2020-03-14	AT
EPI_ISL_419663	2020-03-15	AT
EPI_ISL_419664	2020-03-23	AT
EPI_ISL_419666	2020-03-11	AT
EPI_ISL_419667	2020-03-13	AT
EPI_ISL_420619	2020-03-23	FR
EPI_ISL_417538	2020-03-17	IS
EPI_ISL_428209	2020-04-06	PL
EPI_ISL_429535	2020-03-25	DK
EPI_ISL_429537	2020-03-24	DK
EPI_ISL_406800	2020-01-01	CN
EPI_ISL_406862	2020-01-28	DE
EPI_ISL_418805	2020-03-06	BE
EPI_ISL_418806	2020-03-05	BE
EPI_ISL_428232	2020-03-18	PL
EPI_ISL_428233	2020-03-18	PL
EPI_ISL_428234	2020-03-19	PL
EPI_ISL_428235	2020-03-23	PL
EPI_ISL_428236	2020-03-27	PL
EPI_ISL_429561	2020-03-25	DK
EPI_ISL_429583	2020-03-23	DK
EPI_ISL_416327	2020-01-28	CN
EPI_ISL_416334	2020-02-06	CN
EPI_ISL_417663	2020-03-14	IS
EPI_ISL_417688	2020-03-01	IS
EPI_ISL_418989	2020-03-04	BE
EPI_ISL_416386	2020-01-31	CN
EPI_ISL_427043	2020-03-18	GR
EPI_ISL_428346	2020-04-17	TR
EPI_ISL_428366	2020-03-30	FR
EPI_ISL_428368	2020-04-16	TR
EPI_ISL_417623	2020-03-18	IS
EPI_ISL_428354	2020-03-25	FR
EPI_ISL_429370	2020-03-09	DK
EPI_ISL_418627	2020-03-14	BE
EPI_ISL_418629	2020-03-17	BE
EPI_ISL_418633	2020-03-17	BE
EPI_ISL_418665	2020-03-22	BE
EPI_ISL_429335	2020-03-02	DK
EPI_ISL_429339	2020-03-07	DK
EPI_ISL_417419	2020-03-01	IT
EPI_ISL_417421	2020-03-01	IT
EPI_ISL_417423	2020-03-01	IT
EPI_ISL_417491	2020-03-03	IT
EPI_ISL_429321	2020-03-16	DK
EPI_ISL_429328	2020-03-15	DK

EPI_ISL_429416	2020-03-10	DK
EPI_ISL_430469	2020-02-29	GR
EPI_ISL_406798	2019-12-26	CN
EPI_ISL_416143	2020-02-28	DK
EPI_ISL_417418	2020-03-01	IT
EPI_ISL_417432	2020-03-08	IE
EPI_ISL_417445	2020-02-24	IT
EPI_ISL_417446	2020-02-24	IT
EPI_ISL_417447	2020-02-24	IT
EPI_ISL_417485	2020-03-02	NO
EPI_ISL_417486	2020-03-01	NO
EPI_ISL_429452	2020-03-10	DK
EPI_ISL_410546	2020-01-29	IT
EPI_ISL_421202	2020-03-30	BE
EPI_ISL_433177	2020-04-19	GB
EPI_ISL_434509	2020-04-10	LU
EPI_ISL_410486	2020-02-08	FR
EPI_ISL_411951	2020-02-07	SE
EPI_ISL_421171	2020-03-05	ES
EPI_ISL_421175	2020-03-29	ES
EPI_ISL_422437	2020-03-25	IT
EPI_ISL_422438	2020-03-25	IT
EPI_ISL_422631	2020-03-31	NL
EPI_ISL_434455	2020-03-09	GR
EPI_ISL_434456	2020-03-22	GR
EPI_ISL_434457	2020-03-28	GR
EPI_ISL_434459	2020-03-29	GR
EPI_ISL_434460	2020-03-23	GR
EPI_ISL_434461	2020-03-29	GR
EPI_ISL_434464	2020-03-16	GR
EPI_ISL_434467	2020-03-12	GR
EPI_ISL_434468	2020-03-26	GR
EPI_ISL_434469	2020-03-20	GR
EPI_ISL_434471	2020-03-11	GR
EPI_ISL_434473	2020-03-11	GR
EPI_ISL_434474	2020-03-11	GR
EPI_ISL_434480	2020-03-12	GR
EPI_ISL_434484	2020-03-09	GR
EPI_ISL_434487	2020-04-13	LU
EPI_ISL_434488	2020-04-21	LU
EPI_ISL_422612	2020-04-01	NL
EPI_ISL_422636	2020-03-08	CZ
EPI_ISL_423953	2020-03-18	GB
EPI_ISL_434631	2020-04-08	FR
EPI_ISL_434653	2020-03-24	SE
EPI_ISL_421275	2020-03-18	RU
EPI_ISL_422568	2020-03-28	NL
EPI_ISL_423892	2020-03-17	GB
EPI_ISL_434572	2020-04-14	CZ

EPI_ISL_412912	2020-02-25	DE
EPI_ISL_412971	2020-02-25	FI
EPI_ISL_412973	2020-02-20	IT
EPI_ISL_412974	2020-01-29	IT
EPI_ISL_422266	2020-03-25	GB
EPI_ISL_423662	2020-02-23	GB
EPI_ISL_436980	2020-03-30	DK
EPI_ISL_433016	2020-04-02	GB
EPI_ISL_434366	2020-03-30	BE
EPI_ISL_436999	2020-03-25	DK
EPI_ISL_418000	2020-03-10	PT
EPI_ISL_418006	2020-03-13	PT
EPI_ISL_421653	2020-03-25	LV
EPI_ISL_421654	2020-03-25	LV
EPI_ISL_421655	2020-03-25	LV
EPI_ISL_421656	2020-03-25	LV
EPI_ISL_433639	2020-04-03	GB
EPI_ISL_434358	2020-04-13	BE
EPI_ISL_418021	2020-03-16	PT
EPI_ISL_418027	2020-03-17	PT
EPI_ISL_419386	2020-03-18	PT
EPI_ISL_419387	2020-03-18	PT
EPI_ISL_420312	2020-03-18	NO
EPI_ISL_420313	2020-03-10	NO
EPI_ISL_420358	2020-03-18	BE
EPI_ISL_432332	2020-04-08	GB
EPI_ISL_420294	2020-03-06	SI
EPI_ISL_420295	2020-03-24	SI
EPI_ISL_422906	2020-03-16	NL
EPI_ISL_422922	2020-03-23	NL
EPI_ISL_422926	2020-03-23	NL
EPI_ISL_421743	2020-03-17	LU

EPI_ISL_420464	2020-03-12	GB
EPI_ISL_432438	2020-04-01	GB
EPI_ISL_432446	2020-04-01	GB
EPI_ISL_408430	2020-01-29	FR
EPI_ISL_420401	2020-03-24	BE
EPI_ISL_421735	2020-03-17	LU
EPI_ISL_421736	2020-03-17	LU
EPI_ISL_422717	2020-03-22	NL
EPI_ISL_422731	2020-03-23	NL
EPI_ISL_420129	2020-03-13	ES
EPI_ISL_420134	2020-02-28	NO
EPI_ISL_420135	2020-02-28	NO
EPI_ISL_421448	2020-03-18	PT
EPI_ISL_421450	2020-03-18	PT
EPI_ISL_421456	2020-03-19	PT
EPI_ISL_422740	2020-03-25	NL
EPI_ISL_422756	2020-03-27	NL
EPI_ISL_422793	2020-03-30	NL
EPI_ISL_407073	2020-01-29	GB
EPI_ISL_422679	2020-03-19	NL
EPI_ISL_432000	2020-03-30	GB
EPI_ISL_434657	2020-04-01	SE
EPI_ISL_434658	2020-04-03	SE
EPI_ISL_434659	2020-04-06	SE
EPI_ISL_434660	2020-04-06	SE
EPI_ISL_434663	2020-04-06	SE
EPI_ISL_434664	2020-04-06	SE
EPI_ISL_434666	2020-04-08	SE
EPI_ISL_407079	2020-01-29	FI
EPI_ISL_421507	2020-03-23	FR
EPI_ISL_434672	2020-04-16	SE
EPI_ISL_434675	2020-04-20	SE

EPI_ISL_434676	2020-04-20	SE
EPI_ISL_432233	2020-04-01	GB
EPI_ISL_419176	2020-03-21	FR
EPI_ISL_419187	2020-03-22	FR
EPI_ISL_419254	2020-03-23	IT
EPI_ISL_419255	2020-03-23	IT
EPI_ISL_420136	2020-03-04	NO
EPI_ISL_420137	2020-03-08	NO
EPI_ISL_420138	2020-03-05	NO
EPI_ISL_420139	2020-03-05	NO
EPI_ISL_420141	2020-03-10	NO
EPI_ISL_420143	2020-03-10	NO
EPI_ISL_420145	2020-03-09	NO
EPI_ISL_420146	2020-03-11	NO
EPI_ISL_420147	2020-03-10	NO
EPI_ISL_420148	2020-03-14	NO
EPI_ISL_420150	2020-03-09	NO
EPI_ISL_420151	2020-03-17	NO
EPI_ISL_420152	2020-03-17	NO
EPI_ISL_420153	2020-03-16	NO
EPI_ISL_421467	2020-03-20	PT
EPI_ISL_421471	2020-03-21	PT
EPI_ISL_421472	2020-03-21	PT
EPI_ISL_421474	2020-03-21	PT
EPI_ISL_421477	2020-03-21	PT
EPI_ISL_421482	2020-03-18	PT
EPI_ISL_419173	2020-03-21	FR
EPI_ISL_421492	2020-03-22	PT
EPI_ISL_421493	2020-03-21	PT
EPI_ISL_421494	2020-03-20	PT
EPI_ISL_433459	2020-04-11	GB

AT = Austria, BE = Belgium, CH = Swiss, CN = China, CZ = Czech Republic, DE = Germany, DK = Denmark, ES = Spain, FI = Finland, FR = France, GB = Great Britain, GR = Greece, HU = Hungary, IE = Ireland, IS = Island IT = Italy, LU = Luxemburg, LV = Latvia, NL = Nederland, NO = Norway, PL = Poland, PT = Portugal, RS = Serbia, RU = Russia, SE = Sweden, SI = Slovenia, SK = Slovakia, TU = Turkey.
