# Supplementary material for: Molecular Tracing of SARS-CoV-2 in Italy in the First Three Months of the Epidemic
Source: Viruses. 2020 Jul 24;12(8):798. doi: 10.3390/v12080798 (PMC7472216; doi:10.3390/v12080798)
Supplement: Supplementary file 1 [file viruses-12-00798-s001.zip › Table S3.pdf]

Table S3. Comparison among different demographic models based on Path Sampling (PS) and Stepping Stone (SS) sampling.

Models	PS	SS	Winner
stexpo vs relexpo	-71,87	-71,42	relexpo
stsky vs relexpo_gr	-77,08	-76,06	relexpo_gr
stsky vs relexpo_dt	-68,16	-66,25	relexpo_dt
relsky vs relexpo_gr	20,65	21,55	relsky
relsky vs relcos	27,95	30,20	relsky
relsky vs rellog	15,44	16,91	relsky
relsky vs relexpa	24,36	26,72	relsky
relexpo_gr vs relexpa	3,72	5,17	relexpo_gr
relexpo_gr vs relexpo_dt	-6,15	-5,93	relexpo_dt
relsky vs relexpo_dt	14,49	15,62	relsky
